# Application of Stem Cell-Derived Extracellular Vesicles as an Innovative Theranostics in Microbial Diseases

**DOI:** 10.3389/fmicb.2021.785856

**Published:** 2021-11-30

**Authors:** Hani Keshavarz Alikhani, Bahare Shokoohian, Sama Rezasoltani, Nikoo Hossein-khannazer, Abbas Yadegar, Moustapha Hassan, Massoud Vosough

**Affiliations:** ^1^Department of Regenerative Medicine, Cell Science Research Center, Royan Institute for Stem Cell Biology and Technology, Academic Center for Education, Culture and Research, Tehran, Iran; ^2^Foodborne and Waterborne Diseases Research Center, Research Institute for Gastroenterology and Liver Diseases, Shahid Beheshti University of Medical Sciences, Tehran, Iran; ^3^Gastroenterology and Liver Diseases Research Center, Research Institute for Gastroenterology and Liver Diseases, Shahid Beheshti University of Medical Sciences, Tehran, Iran; ^4^Experimental Cancer Medicine, Institution for Laboratory Medicine, Karolinska Institute, Stockholm, Sweden

**Keywords:** stem cells, infectious disease, antimicrobial agents, bacterial EVs, Viral EVs, MSC-derived EVs, extracellular vesicles

## Abstract

Extracellular vesicles (EVs), as nano-/micro-scale vehicles, are membranous particles containing various cargoes including peptides, proteins, different types of RNAs and other nucleic acids, and lipids. These vesicles are produced by all cell types, in which stem cells are a potent source for them. Stem cell-derived EVs could be promising platforms for treatment of infectious diseases and early diagnosis. Infectious diseases are responsible for more than 11 million deaths annually. Highly transmissible nature of some microbes, such as newly emerged severe acute respiratory syndrome coronavirus 2 (SARS-CoV-2), drives researcher’s interest to set up different strategies to develop novel therapeutic strategies. Recently, EVs-based diagnostic and therapeutic approaches have been launched and gaining momentum very fast. The efficiency of stem cell-derived EVs on treatment of clinical complications of different viruses and bacteria, such as SARS-CoV-2, hepatitis B virus (HBV), hepatitis C virus (HCV), human immunodeficiency virus (HIV), *Staphylococcus aureus*, *Escherichia coli* has been demonstrated. On the other hand, microbial pathogens are able to incorporate their components into their EVs. The microbe-derived EVs have different physiological and pathological impacts on the other organisms. In this review, we briefly discussed biogenesis and the fate of EVs. Then, EV-based therapy was described and recent developments in understanding the potential application of stem cell-derived EVs on pathogenic microorganisms were recapitulated. Furthermore, the mechanisms by which EVs were exploited to fight against infectious diseases were highlighted. Finally, the deriver challenges in translation of stem cell-derived EVs into the clinical arena were explored.

## Introduction

### What Are the Extracellular Vesicles?

Extracellular vesicles as “pro-coagulant dust” were identified by Wolf from blood platelets in Wolf (1967), and then in Pan and Johnstone (1983) were among the first scientists who described EVs (Pan and Johnstone, 1983). EVs are a heterogeneous group of vesicles containing different cargos (Abels and Breakefield, 2016). They can transfer different biomolecules such as lipids, proteins, and nucleic acids between different cells, distinguishing their significant role in cell-cell communications (Raposo and Stoorvogel, 2013). EVs are generated and released by almost all types of cells and are classified as exosomes, macrovesicles, and apoptotic bodies (Gurunathan et al., 2021; Pournaghi et al., 2021). Also, ectosomes, shedding vesicles, and microparticles are other types of EVs involved in inter/intra cellular communications (Gurunathan et al., 2019). EVs have attracted tremendous attention from both basic and clinical fields of study during the last decade due to their putative and significant role in several physiological and pathological processes (Jadli et al., 2020). EVs have been isolated from different body fluids such as blood, urine, tears, saliva, etc. (Akers et al., 2013). Some disorders such as inflammatory diseases can modify the EVs and change their numbers, content, composition, and function (Knijff-Dutmer et al., 2002). It has been shown that microbial infections can change the production and release process of EVs in infected cells (Rodrigues et al., 2018). Moreover, some EVs derived from immune cells can play a key role in induction of inflammation (Spencer and Yeruva, 2021).

### Types of Extracellular Vesicles

Macrovesicles (MVs), also known as microparticles, are small membranous vesicles released from almost all cell types including mesenchymal stem cells, endothelial cells, some immune cells, etc. (Cocucci et al., 2009). Their size is ranging from 100 to 1000 nm, and they are formed by direct outward blebbing and pinching of the cell membrane. The production of EVs is regulated by physiological and/or pathological processes (Lynch and Ludlam, 2007; Sedgwick and D’Souza-Schorey, 2018). Initially, MVs are considered as cell debris, however, they recently were recognized as mediators of inter/intra cellular communication tools (Camussi et al., 2010). MVs can carry various bioactive molecules such as cytokines and chemokines, which highlight their antimicrobial potential and also their role in host defense against pathogenic microorganisms (Timár et al., 2013).

Apoptotic cell-derived EVs (ApoEVs) are another class of EVs released from apoptotic cells and contain cell organelles and nuclear materials (Gurunathan et al., 2019). ApoEVs are divided into two subtypes, including large apoptotic bodies (ApoBDs) with a diameter range of 1000–5000 nm and small apoptotic microvesicles (ApoMVs) with <1000 nm (Caruso and Poon, 2018). ApoEVs are important because they accelerate apoptotic cell clearance and also have a role in intercellular communication and immune modulation (Zitvogel et al., 2010; Poon et al., 2014). ApoEVs act as a key regulator of antigen presentation process, antimicrobial immunity against pathogens, and modulator of the dendritic cells’ response against viral infections (Winau et al., 2006; Schiller et al., 2008; Caruso and Poon, 2018).

Exosomes (30-150 nm in diameter) are the third group of EVs isolated from a variety of body fluids and released by the fusion of multivesicular bodies (MVBs) with the plasma membrane (Simons and Raposo, 2009; Babaei and Rezaie, 2021; Mobarak et al., 2021). Some exosomes are generated and released from various cells in response to different stimuli, but others are continuously produced and released (Mathivanan et al., 2010). They contain various types of cargo molecules which are engaged in the biogenesis and transportation ability of exosomes (Zhang et al., 2019). Exosomes have been implicated in a variety of biological functions, including elimination of old and disused biomolecules (Harding et al., 2013), involvement in tumor progression especially in angiogenesis and metastases (Rak, 2010; Hood et al., 2011), antigen presentation (Bobrie et al., 2011), differentiation of some immune cells to modulate immune responses (Zhang and Grizzle, 2011), and facilitating the spread of some pathogenic microbes or elimination of microbes through interaction with recipient cells (Furuyama and Sircili, 2021; White et al., 2021).

### Isolation Methods of Extracellular Vesicles

Several isolation methods are currently developed for the isolation and purification of EVs in bulk (Witwer et al., 2013). Sequential centrifugation and ultracentrifugation are the conventional methods to isolate EVs in cell culture media or body fluids (Théry et al., 2006). Gradient ultracentrifugation based on sucrose density is also used to minimize protein contamination (Tauro et al., 2012). Chromatography is another tool that can be employed to purify exosomes based on their size and dimensions or surface markers such as CD9, CD63, CD81, and EpCAM (Böing et al., 2014; Oksvold et al., 2015). Once isolated, the purified EVs are characterized. Currently, several methods are developed to analyze the EVs and their content for both research and clinical purposes. These methods include transmission and scanning electron microscopy (TEM and SEM), atomic force microscopy (AFM), dynamic light scattering (DLS), nanoparticle tracking analysis (NTA), resistive pulse sensing (RPS), flow cytometry, fluorescence-activated cell sorting (FACS), enzyme-linked immunosorbent assay (ELISA), microfluidics, and electrochemical biosensor-based devices (Théry et al., 2006; Woo et al., 2016).

### Contents of Extracellular Vesicles

The content, or cargo, of EVs varies and extremely depends on the parental cells and recently new databases including ExoCarta, Vesiclepedia, exoRBase, EVmiRNA, and EVpedia were developed to classify them (Liu et al., 2019). These databases provide information about the content of EVs such as lipids, proteins, miRNAs, and other components. Also they provide useful information about the isolation and characterization of EVs (Jadli et al., 2020). The proteome of the EVs is affected by their biogenesis. For example, ESCRT proteins (Alix, TSG101, HSC70, and HSP90β) regulate the biogenesis and transportation of some EVs and thus these proteins are expected to be found in EVs regardless of the type of the originating cells (Théry et al., 2001; Van Niel et al., 2006). Therefore these proteins can be used as marker for the detection and characterization of EVs (Doyle and Wang, 2019). Some tetraspanin families of proteins such as CD63, CD9, and CD81 are commonly found in EVs and also used as marker proteins both for the detection and purification of EVs (Witwer et al., 2013). Along with the exosome and some EVs surface markers, EVs carry certain biomolecules such as mRNA, miRNA (Zhou J. et al., 2013), cytokines, and antigen presentation molecules (MHC-I, MHC-II) (Gutiérrez-Vázquez et al., 2013) which contribute to the physiological and pathological function of exosomes (Dini et al., 2020). Microbial EVs contain different cargoes based on their origin. The EVs of Gram-negative bacteria contain cytoplasmic proteins, nucleic acids, virulence factors (e.g., toxins), peptidoglycan, and inner membrane. The EVs of Gram-positive bacteria contain membrane-associated virulence proteins, fatty acids, lipoteichoic acid, phospholipids, and some components similar to the Gram-negative EVs (Yu et al., 2018; Bose et al., 2020).

### Common Uses of Extracellular Vesicles

As mentioned above, EVs are mainly responsible for inter/intracellular communications. It was shown that EVs could interact with target cells and therefore they make an impact on cell physiology, phenotype, and function (Simons and Raposo, 2009; Mardpour et al., 2018). Also they can mediate the horizontal transfer of genetic materials (Natasha et al., 2014). Due to the widespread and cell-specific availability of some types of EVs, particularly exosomes, in almost all body fluids, they can be considered as biomarkers (Zhang et al., 2019). Moreover, EVs can be used as delivery vehicles for the efficient transfer of biological therapeutic agents across different biological barriers to desired cells (Haney et al., 2015). In addition, EVs can be applied in regenerative medicine, tissue engineering and cell homeostasis (Gurunathan et al., 2021). They play critical roles in immunoregulation, including antigen presentation, immune activation, immune suppression, and also immune tolerance via exosome-mediated inter/intracellular communications. They also play pivotal role in the host defense against viral and microbial infections (Gurunathan et al., 2021). Documented evidence has shown that host cells-derived EVs and even EVs derived from bacteria can mediate the crosstalk between pathogen and innate immune cells, and thus modulate the innate immune responses of the host (Munich et al., 2012).

In this review article, we first discussed the biogenesis and the fate of EVs. Then, EV-based therapy was described and recent developments in understanding the potential application of stem cell-derived EVs in infectious diseases were recapitulated. In addition, the mechanisms by which EVs were exploited to fight against infectious diseases were highlighted. Finally, the deriver challenges that exists in the translation of stem cell-derived EVs into the clinical arena were explored.

## Biogenesis and the Fate of Extracellular Vesicles

### Biogenesis

**FIGURE 1:**
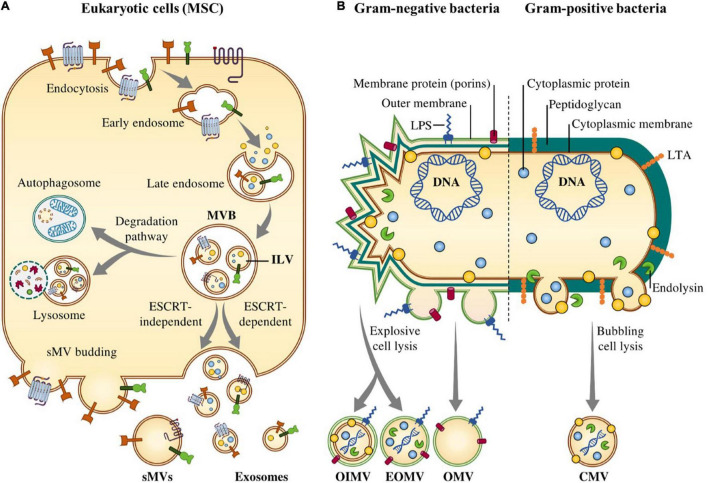
Biogenesis of Extracellular vesicles (EVs) in eukaryotic cells and Gram-negative and –positive bacteria. **(A)** In eukaryotic cells, biogenesis of EVs consists of three consecutive steps, including (i) formation of early endosome by invagination of the plasma membrane; (ii) formation of late endosome and then MVBs; and finally (iii) fusion of MVBs with the plasma membrane and release of the vesicular contents by ESCRT-dependent and –independent mechanisms. **(B)** In the Gram-negative bacterial extracellular vesicles, known as outer membrane vesicles mainly originate from the outer membrane of bacterial envelope. Three potential biogenesis mechanisms have been suggested including the blebbing of the outer membrane of the bacterial envelope (OMVs), the formation of outer-inner membrane vesicles (OIMVs) and the formation of explosive outer membrane vesicles (EOMVs). Gram-positive bacteria **(B)** lack an outer membrane and also have a thick peptidoglycan cell wall outside of the cell membrane which convey the assumption that membrane-derived vesicles could not escape such large barriers but EVs may be forced through the wall by turgor pressure after release from the plasma membrane. In addition, cell wall-modifying enzymes facilitate the released of EVs.

The biogenesis of the exosomes is well-defined as compared to the other types of EVs. The biogenesis of exosomes is a multistep biological process regulated through different signaling pathways (Abels and Breakefield, 2016). Biogenesis of exosomes initiates with the formation of early endosomes followed by second inward budding of the endosomal membrane which leads to the formation of the late endosomes. Late endosomes or intraluminal vesicles (ILVs) follow the endocytic pathway for the generation of exosomes (Sluijter et al., 2014). In the final stage, the generated ILVs are released as exosomes into the extracellular space via exocytosis (Jadli et al., 2020). Some endogenous molecules such as small GTPase Ral (Hyenne et al., 2018) and adiponectin/T-cadherin (Obata et al., 2018) and also some microbes including viral infections and Gram-positive and Gram-negative bacterial infections as extrinsic factors can influence the biogenesis of exosomes (Crenshaw et al., 2018). For the biogenesis of exosomes, different protein sorting mechanisms have been identified, among them endosomal sorting complex transport (ESCRT)-dependent pathway (Frankel and Audhya, 2018) and ESCRT-independent pathway (Babst, 2011) are two widely explained mechanisms (Figure 1).

#### Endosomal Sorting Complex Transport-Dependent Mechanism

The ESCRT-dependent mechanism is well-characterized and comprised of many proteins arranged into four proteins complexes including ESCRT-0, –I, –II, and –III. Some proteins such as VPS4, VTA1, and ALIX are associated with these protein complexes. The ESCRT-dependant exosome biogenesis is initiated by recognition and sequestration of ubiquitinated proteins via ubiquitin-binding subunits of ESCRT-0. Then, the ESCRT-0 interacts with the ESCRT-I and –II complexes and all of them will combine with ESCRT-III, a protein complex that is contributed to enhance budding processes. Finally, following cleaving the buds to form ILVs, the ESCRT-III complex disassociates from the MVB membrane with energy supplied by the sorting protein Vps4 (Ren et al., 2008).

#### Endosomal Sorting Complex Transport-Independent Mechanism

While ESCRT pathway is the most important mechanism for exosome formation, some EVs such as MBV and ILV are also formed in an ubiquitin-independent way called CRT-independent pathways (Babst, 2011). Heparan sulfate promotes exosome biogenesis through syntenin. Syntenin serves as an intermediate between ESCRT-I and ESCRT-III and is involved in the budding processes (Addi, 2019). Another ESCRT-independent exosome formation was described in the oligodendroglial cells. These cells secret exosomes containing proteolipid protein (PLP) which depends on the depletion of neutral sphingomyelinases (nSMase). Furthermore, tetraspanin-enriched microdomains (TEMs) full of CD81 particles are regarded to be another ESCRT-independent pathway of protein sorting into ILVs (Garín et al., 2007).

### Extracellular Vesicles Secretion

To release exosomes, multiple cellular steps are required to be completed including formation of MVBs, transportation of MVBs to the plasma membrane, and fusion of MVBs with the plasma membrane. Several molecules have been engaged in these processes (Rabinovich et al., 2000). MVB can either fuse with a lysosome to degrade their cargo or fuse with the plasma membrane, leading to exosome release. ISGylation, a post-translational ubiquitin-like modification, is one of the signals that regulates the MVBs’ fate. ISGylation of MVB proteins promotes fusion of MVBs with lysosomes, which promoting the exosome release (Chen et al., 2003).

### Mechanisms of Exosome Uptake

Following the release and secretion of EVs to the extracellular environment, the EVs’ ability to interact with recipient cells and capacity to deliver their contents such as proteins, lipids, and nucleic acids into the recipient cells can determine the role of EVs in physiological and pathological processes (Jadli et al., 2020). Several mechanisms have been introduced for the uptake of exosomes, including phagocytosis, macropinocytosis, clathrin-mediated endocytosis (CME), caveolin-dependent endocytosis (CDE), and plasma membrane fusion (Tamura et al., 2016; Sun et al., 2018). Phagocytosis is in charge of the internalization of bacteria, EVs, and others components. Toll-like receptors (TLRs), scavenger, and complement receptors, as specific targets on the cell surface, participate in invaginations around the material intended for internalization (Wei et al., 2021). Macropinocytosis is another mechanism characterized by plasma membrane ruffling and is induced by growth factors or other signal stimulations. Membrane ruffles form a cup-like structure that seals at its distal tips to construct a relatively large endosome (Canton, 2018). The resulting vesicles contain extracellular fluid and small particles (Wei et al., 2021). CME is a receptor-mediated endocytic process used to transport a wide range of cargo molecules from the cell surface to the interior. Clathrin and adaptor protein 2 (AP2) complexes are necessary for the formation of clathrin-coated vesicles and then the internalization of cargo molecules (Palmirotta et al., 2018). CDE requires the caveolin, a dimeric protein, in the plasma membrane to facilitate the raft-mediated endocytosis (Melo et al., 2015). CDE is another mechanism for the uptake of EVs, but the precise mechanism of internalization may differ depending on the type of EVs and recipient cells (Nanbo et al., 2013). Fusion is the last mechanism of EVs internalization, which enables EVs membrane to directly merge with the cell plasma membrane and transfer cargo molecules to recipient cells (Jadli et al., 2020).

## Extracellular Vesicles-Based Therapy of Microbial Diseases

### Role of Extracellular Vesicles in Bacterial and Viral Infections

Eukaryotic cells generate a heterogeneous group of EV subtypes, recognized by biogenesis mechanism and their size (Sedgwick and D’Souza-Schorey, 2018; Théry et al., 2018). EVs have been observed in all body fluids of humans (Raposo et al., 1996; Lässer et al., 2011; Street et al., 2012; Roberts and Kurre, 2013; Hiemstra et al., 2014) and are generated by various cell types (Raposo et al., 1996; Barry et al., 1998; Van Niel et al., 2001; Lai and Breakefield, 2012; Peinado et al., 2012). EVs are responsible for developing the functional range of the bioactive molecules secreted by cells, improving their stability, and boosting their ability to achieve better localized concentrations (Liu et al., 2019; Guo et al., 2021). EVs have an important role in delivery and orchestration of antimicrobial responses from host immune system. In infectious diseases, the cells such as epithelial cells and macrophages have the first contact with the EVs of pathogens that containing bioactive molecules and induce host pro-inflammatory responses (Hui Winnie et al., 2018; Li et al., 2018; White et al., 2021). The stimulated immune cells during infections may produce EVs that convey antimicrobial agents (Kesimer et al., 2009; Hu et al., 2013; Timár et al., 2013) or play decoy roles to protect host cells by binding and coating toxins secreted from bacterial pathogens (da Cruz et al., 2020) (Table 1). Production of EVs by a variety of host cells may be enhanced during infectious diseases (da Cruz et al., 2020). Different types of microorganisms and viruses have all been reported to directly stimulate host EVs secretion (Kim et al., 2012; Antwi-Baffour et al., 2020; Mehanny et al., 2020). The enhancement of host EVs secretion has been shown following an extracellular challenge of alveolar epithelial cells with heat-sacrificed bacteria, providing that the activator of this response was bacterial CpG DNA that bond to endosomal receptors of TLR9 (Keller et al., 2020). Host endothelial cells and macrophages infected by bacterial agents are similarly promoted to release EVs (Bhatnagar et al., 2007; Hui Winnie et al., 2018; Li et al., 2018). Besides increasing the production of EVs, infections can alter the contents of EVs produced by host cells (Bhatnagar et al., 2007). MSCs-derived EVs promote healing process in diabetic foot by loading some bioactive molecules including growth factors, nucleic acids, and proteins. Also, as a vehicle for non-bioactive substances like antibiotics can inhibit the bacterial growth and accelerate improvement in the diabetic wound repair in bacteria-associated diabetic foot ulcers (Raghav et al., 2021).

**TABLE 1:** Current biomedical applications of Extracellular vesicles.

EVs in Biomedicine			References
Bacterial EVs	EVs as vaccine candidate (outer membrane vesicles (OMVs))		Balhuizen et al., 2021
	EVs as anticancer drugs (membrane vesicles (MVs))		Chronopoulos and Kalluri, 2020
	Role of EVs in antibiotic resistance and biofilm formation		Bitto et al., 2017
	EVs as immune modulator factors		Riley et al., 2013
	EVs as infection biomarkers		Deng et al., 2020
Viral EVs	EVs can facilitate viral infection		Nolte-‘t Hoen et al., 2016
	EVs can intensify inflammatory responses and deflagrate antiviral activities		Urbanelli et al., 2019
MSCs-derived EVs	Immunomodulatory functions of EVs derived from MSCs		Dabrowska et al., 2021
	MSCs-derived EVs for treatment of COVID-19 and other viral infections		Golchin et al., 2020
	Secretion of antimicrobial peptides and proteins (AMPs) loaded in EVs	Cathelicidin LL-37	Sandra Tjabringa et al., 2005
		Human β-defensin-2 (hBD-2)	Alcayaga-Miranda et al., 2017
		Hepcidin	Lombardi et al., 2015
		Lipocalin-2 (Lcn2)	Wang Q. et al., 2019
		Indoleamine 2, 3-dioxygenase (IDO)	Schroten et al., 2001
		Interleukin-17 (IL-17)	Yang et al., 2013
	Decoy EVs provide protection against bacterial toxins		Keller et al., 2020

#### Bacterial Extracellular Vesicles

It is now well recognized that most bacteria produce soluble products such as metabolites, quorum sensing peptides, nucleic acids, proteins, and bacterial EVs (BEVs) that allow their communication with each other and host cells (Hughes and Sperandio, 2008; Tulkens et al., 2020). Interestingly, both beneficial and pathogenic bacteria release BEVs. BEVs carry various molecules such as proteins, peptidoglycan, enzymes, toxins, polysaccharides and DNA/RNA molecules (Riley et al., 2013; Kaparakis-Liaskos and Ferrero, 2015). Different BEVs have various structures and even various molecular cargo compounds. These differences may be due to various growth conditions, several biogenesis pathways, the unique membrane envelope structure of the parental bacterium which they emanate from, and also the genetic content of the paternal bacterial strain (Toyofuku et al., 2019). BEVs display high stability to different temperatures and treatments, and regarded safe because they are not able to replicate *in vitro* and *in vivo* conditions. They carry several immunogenic surface and membrane related components of their parental strains (Kaparakis-Liaskos and Ferrero, 2015). Based on the originating strains, BEVs can promote both humoral and cellular immunity and together with their nanoparticulate character, provide them with their own adjuvanticity, BEVs are capable to increase T-cell reactions to antigens (Chronopoulos and Kalluri, 2020).

##### Application of outer membrane vesicles and vaccine development

Gram-negative bacteria pursue two important routes for BEVs production. The primary pathway is blebbing of the outer membrane of the bacterial envelope, producing OMVs; and the other pathway requires explosive cell lysis forming outer-inner membrane vesicles (OIMVs) and explosive outer membrane vesicles (EOMVs) (Figure 1; Toyofuku et al., 2019; Tulkens et al., 2020). The blebbing process of the membrane giving rise to OMVs happens through a disruption of crosslinks between the outer membrane and the underlying peptidoglycan cell wall layer (Chronopoulos and Kalluri, 2020). Actually, the Gram-negative bacterial cell wall comprises a thin layer of peptidoglycan in the periplasmic environment between two membrane bilayers; the cytoplasmic and outer membranes (Toyofuku et al., 2019; Chronopoulos and Kalluri, 2020). The outer membrane includes lipopolysaccharides (LPS) or endotoxin on its outer leaflet and various membrane-bound channels and protein-like porins that simplify non-vesicle mediated transport. Reflecting this envelope structure, Gram-negative BEVs consist of an outer membrane with an interior leaflet of phospholipids and an exterior leaflet of LPS that is known to engage TLR4 (Toyofuku et al., 2019; Tulkens et al., 2020). BEVs of Gram-negative bacteria contain high concentration of different outer membrane proteins, such as ompA and encapsulated periplasmic luminal compounds. Nevertheless, the existence of cytoplasmic cargo including virulence molecules and nucleic acids is debated and dependent on the certain biogenesis pathways of the OMV, OIMVs, and EOMVs (Toyofuku et al., 2019; Tulkens et al., 2020). The endotoxicity of BEVs can be simply modified through genetic engineering methods. Furthermore, BEVs from specific commensal, or beneficial and potentially probiotic bacterial species may excrete therapeutic effects. In future, BEVs may be applied in cancer immunotherapy to elicit durable antitumor immune response or act as anti-cancer vaccines. In a recent study, the applicability of BEVs in cancer immunotherapy or cancer vaccines was reported showing that systemic intravenous administration of Gram-negative BEVs from the genetically modified *Escherichia coli* msbB^–/–^ has a directed tropism for tumor site and a notably capability of inducing long-term antitumor immune responses through the secretion of CXCL10 and INFγ that can completely eradicate tumors without considerable adverse consequences (Kim et al., 2017).

##### Application of membrane vesicles

Gram-positive bacteria also release nano-sized cytoplasmic membrane vesicles (CMVs), or so-called as MVs, through endolysin-triggered bubbling cell death into the extracellular environment either in a constitutive manner or in a regulated manner (Brown et al., 2015; Toyofuku et al., 2019). Gram-positive bacteria lack the entire outer membrane and possess a much thicker peptidoglycan layer or cell wall. The Gram-positive cell wall is connected to glycan polymers that can be covalently linked to peptidoglycan [as wall teichoic acids (WTAs)], or anchored in the cell membrane in the case of glycolipids such as lipoteichoic acids (LTAs) which can interact with TLR2 (Brown et al., 2015; Toyofuku et al., 2019; Tulkens et al., 2020). Similar antitumor effects were also seen for the Gram-positive BEVs originated from *Staphylococcus aureus* and *Lactobacillus acidophilus* (Chronopoulos and Kalluri, 2020). There is also an enormous interest in applying genetic engineering methods to manipulate bacteria and subsequently purify recombinant BEVs for utility as vaccines against some cancers (Chronopoulos and Kalluri, 2020).

##### Role of extracellular vesicles in antibiotic resistance and biofilm formation

The localization of chromosomal DNA in BEVs from different Gram-negative pathogenic bacteria such as *Salmonella typhimurium* is often extraluminal with smaller amounts settled in the intraluminal locations (Bitto et al., 2017). Sequencing of the intraluminal BEV DNA has been reported to be enriched in certain regions of the bacterial chromosome involved in pathogenicity and virulence capacity, stress response and antibiotic resistance as well as metabolic pathways. There is still a matter of controversy whether extraluminal or surfaces-associated BEV DNAs versus intraluminal ones render different types of actions. One can assume a probable role for external DNA in biofilm formation versus a role for internal BEV DNA in intercellular crosstalk and horizontal gene transfer (HGT) of virulence-associated markers and antibiotic resistance encoding genes (Bitto et al., 2017).

##### Bacterial extracellular vesicles contribute to immune response

Bacterial extracellular vesicles contain several microbe-associated molecular patterns (MAMPs) or pathogen-associated molecular patterns (PAMPs) such as peptidoglycan, lipoproteins, LPS and bacterial DNA/RNA. The MAMP content of BEVs enables them to interact with host pattern recognition receptors (PRR) in different types of host cells to induce immune tolerance, or confer protective immunity, and even host cell damage (Riley et al., 2013). The immunomodulatory effects of BEVs mainly depend on the particular parental bacterium and its association with the host. For example, BEVs from some pathogenic bacteria are capable of worsening the infection by suppressing host immune responses (Peek and Blaser, 2002; Lee Hannah et al., 2007), or induce overwhelming immune responses leading to sepsis (Shah et al., 2012). In opposite, BEVs from beneficial or commensal bacteria in the gut can promote immunological maturation and tolerance to confer protection from sepsis (Shen et al., 2012; Kang et al., 2013). In addition, a number of cell surface TLRs, prominently TLR2 and TLR4, can recognize extraluminal ligands of BEV such as LPS, lipoarabinomannan, peptidoglycan and LTA macromolecules (Prados-Rosales et al., 2011; Zhao et al., 2013; Athman et al., 2015; Gu et al., 2019). Also, both nucleotide-binding oligomerization domain-containing protein 1 (NOD1) and NOD2 are engaged in sensing peptidoglycans that are available in BEV contents produced by pathogenic and symbiotic bacterial strains (Kaparakis et al., 2010; Thay et al., 2014; Bitto et al., 2018; Cañas et al., 2018). In addition, intraluminal BEV DNA/RNA may be recognized via DNA/RNA sensing receptors. After endocytosis, BEV RNA cargo may be recognized via endosomal TLRs such as TLR3 and TLR7. In a similar fashion, RNAs rendered into the cytoplasm after fusion of BEVs with the cell plasma membrane may activate cytosolic RNA detectors like RIG-I-like receptors (Tsatsaronis et al., 2018). Similarly, BEV DNA cargo may be sensed by endosomal TLR9 or cytosolic DNA sensing cyclic GMP-AMP synthase stimulator of the interferon genes cascade. Overall, PRR stimulation promotes the activation of transcription factors and kinases that result in the secretion of chemokines and other cytokines leading to the immune cells recruitment and regulation of costimulatory factors normally associated with acquired immune response (Riley et al., 2013).

##### Extracellular vesicles as infection biomarkers

As mentioned above, EVs are found in various body fluids like blood, urine and saliva. EVs contain different biomolecules, which can be used as novel biomarkers for a variety of human diseases and cancers. Since they can be obtained by minimally invasive biopsy procedures, thus they would be very useful biomarkers for diagnosis (Lee et al., 2018; Min et al., 2019). As researchers begin to figure out the distribution pattern and composition of EVs during infectious diseases, new biomarkers can be introduced that can provide the possibility for the development of EV-based diagnostics (Tulkens et al., 2020). For instance, serum EVs can used to show biofilm-associated infections to support a rapid detection (Deng et al., 2020). Further understanding of the biology of EVs can provide possible clues to protect infectious diseases and early detection.

#### Viral Extracellular Vesicles

Viral EVs are generated by virus-infected cells and are considered to be engaged in inter/intracellular connection between infected and uninfected cells. Viral agents, especially oncogenic viruses and viruses that can develop chronic infections can regulate the EVs generation and the content. Viruses are defined by the virologists of last century as “submicroscopic infectious agents that can replicate only inside the living cells of an organism”. EVs do not fall under this description, because contrary to their similarity to viruses in many aspects, they are basically distinct, as they are non-replicative particles. Nevertheless, current virology has distanced itself from such an out-dated description of virus by new terms of defective and non-infectious particles. Thus, EVs produced by virus-infected cells that contain different viral proteins and some parts of viral genomes can fall under the description of non-infectious viral agents (Nolte-‘t Hoen et al., 2016). Moreover, there is a resemblance between biogenesis of virions and EVs. EVs and virus particles are altogether released by infected cells and share the routes for biogenesis at the plasma membrane (Colombo et al., 2014). Regardless of what described, it is essentially difficult to discriminate between EVs that deliver viral proteins, viral genomic fragments and host proteins and enveloped viral particles that carry the similar contents (Nolte-‘t Hoen et al., 2016).

##### Effects of extracellular vesicles on viral pathogenesis

It was shown that cells infected with enveloped or non-enveloped viruses produce EVs that carry viral constituents. The preparation of viral particles may not be completely pure and are almost mixed with various types of EVs, and even some of these EVs may be either indiscernible from defective viruses. Due to their close biogenesis routes, EVs and viral particles may be near relatives, however only viruses can replicate inside the cells. Notably, EVs produced via infected cells are not neutral, as they may facilitate virus spread and viral infection or increase antiviral responses (Nolte-‘t Hoen et al., 2016). For instance; numerous HIV RNAs and proteins have been identified in EVs produced from HIV-infected cells (Narayanan et al., 2013). The involvement of EVs in viral infection has already been described for many viruses, including rabies, coronaviruses, HCV, HIV, HPV, HSV, dengue, HTLV-1, Zika, West Nile Epstein Baar virus, influenza virus, and SFTS (Martins and Alves, 2020). Deciphering the EVs structure produced by infected cells, characterizing their cargo, and understanding the accurate strategy by which they affect viral infection are necessary for basic virology and therapeutic applications as well (Nolte-‘t Hoen et al., 2016).

##### Effects of viral extracellular vesicles on host immunity

The process of infection may alter the contents of cells-derived EVs and change the ratios of host RNAs and proteins inside them (Nolte-‘t Hoen et al., 2016). Upon infection process, EVs can intensify inflammatory responses and deflagrate antiviral activities (Urbanelli et al., 2019), also can mediate the crosstalk between immune cells and other cells (Isola and Chen, 2017; Rezaie et al., 2021). Those EVs which can be transferred between the immune cells may transmit signals affecting the chemokines or cytokine secretion level, and some EVs can directly activate antigen presentation (Lindenbergh and Stoorvogel, 2018). Also, EVs carry different cytokines and cytokine-associated RNAs that may trigger the generation of target molecules in recipient cells, contributing to antiviral responses (Urbanelli et al., 2019). Moreover, EVs produced by infected cells were able to trigger other cell types, as observed when EVs produced by U937 macrophages, contaminated with DENV-2, activated endothelial cells (Velandia-Romero et al., 2020). Also, EVs secreted from airway epithelial cells infected by respiratory syncytial virus (RSV) can enhance the expression of regulatory small RNAs and may activate chemokine and cytokine secretion in monocytes without being exposed to infective particles (Chahar et al., 2018).

Further, EVs are capable to mediate the severity of disease though increasing the secretion of pro-inflammatory cytokines associated with several infectious diseases. EVs derived from bronchoalveolar fluid of mice infected with H5N1 influenza virus displayed enrichment with miR-483-3p, which stimulates innate immunity in pneumocytes proposing the involvement of EVs in the inflammatory pathogenesis of H5N1 virus (Maemura et al., 2020). In addition, in dengue hemorrhagic fever, EVs were observed to play an important role in the disease development (Mishra et al., 2019).

##### Immune cell-derived extracellular vesicles and antiviral effects

Extracellular vesicles can interact with each other and with viruses *in vivo* either directly or indirectly though modulating the host responses, therefore they are engaged in a “War and Peace” scenario between host and viruses (Lisco et al., 2009; Bhattarai et al., 2015). In opposite, EVs containing viral proteins can be profitable to the host cells. For instance, they can present viral antigens to DCs to facilitate triggering activation of adaptive immunity. Therefore, EVs produced within viral infection may demonstrate either proviral or antiviral features. It has been shown that T cells could release EVs comprising HIV receptor CD4. Such EVs can directly bind to viral particles, thereby reducing the load of virions that would otherwise infect other CD4^+^ T cells (de Carvalho et al., 2014). Thus, further investigations are required to uncover the exact roles of EVs in antiviral immune responses in order to direct EVs engineering that may exhibit robust antiviral potentials.

### Host Extracellular Vesicles as Antimicrobial Responses

Host EVs produced by immune cells have been demonstrated to elicit strong antimicrobial effects in *in vitro* and *in vivo* conditions (Timár et al., 2013; Wang C. et al., 2019). These antimicrobial potencies are contributed to various bacteriostatic and bactericidal compounds present in the EVs cargo (Table 1) (Timár et al., 2013; Hiemstra et al., 2014). For instance, EVs obtained from human urinary tract are usually enriched in proteins with immune functions, such as bacteriostatic proteins like mucin-1, fibronectin, CD14, and also bactericidal proteins such as calprotectin, dermcidin and lysozyme C (Hiemstra et al., 2014). Moreover, such EVs hindered the growth of probiotic and uropathogenic strains of *E. coli* as well as its laboratory-adopted strains, and mediated the bactericidal functions through a lytic process (Hiemstra et al., 2014). Also, EVs are assumed to stabilize bioactive components like RNAs, and affect the non-lethal pathways of controlling microbial behaviors (Liu et al., 2016). EVs released from the host cells upon exposure to pathogenic microbes may also be able to safeguard cells from microbial assaults via efficient imitating the targets of their toxins and functioning as decoys (Keller et al., 2020).

#### Immunomodulatory Functions of Extracellular Vesicles Derived From Mesenchymal Stem Cells

Mesenchymal stem cells (MSCs) are multipotent stem cells derived from mesoderm and have the ability to differentiate into a variety of cells. They can be easily obtained from many sources including mature tissues such as bone marrow (BM), peripheral blood (PB), adipose tissues (AT) and neonatal birth-related tissues including amniotic fluid (AF), Warton jelly (WJ), umbilical cord (UC), placenta (PL), and cord blood (CB) (Seo et al., 2019). These heterogeneous cells possess significant immunomodulatory and protective characteristics (Dabrowska et al., 2021). They are also modulate immune cell responses and produce various inflammatory mediators by which they can regulate both innate and adaptive immune responses. When the responses of some immune cells such as macrophages, natural killer (NK) cells, DCs, B and T cells are exaggerated, MSCs can repress their proliferation, differentiation, and activation to modulate the immune response (Ren et al., 2008). These immunomodulatory effects are exerted through the production of several soluble mediators such as nitric oxide (NO), indoleamine 2, 3-dioxygenase-1 (IDO-1), transforming growth factor-β1 (TGF-β1), interleukin-10 (IL-10), prostaglandin-E2 (PGE2), and hepatocyte growth factor (HGF) to the microenvironment (Puissant et al., 2005). Such immunomodulatory effects of MSCs might be associated with EVs which they release to the environment. It has been shown that EVs released from MSCs can inhibit the proliferation and differentiation of B lymphocytes in a dose-dependent manner (Budoni et al., 2013). Also, EVs produced by murine BM-MSCs inhibit the proliferation of T lymphocytes and modulate the adaptive immune system in mice via inducing of apoptosis in the activated T lymphocytes. This increases the number of regulatory T cells, and enhancing the secretion of anti-inflammatory cytokines such as IL-10 and TGF-β1 (Mokarizadeh et al., 2012). Notably, it has been reported that galectin-1 and programmed death receptor ligand (PD-L1) were present on the surface of EVs derived from MSCs (Garín et al., 2007). Endogenous galectin-1 induces apoptosis in the activated T lymphocytes and provoke the maturation of regulatory T lymphocytes (Rabinovich et al., 2000). PD-L1, on the other hand, is a ligand of the PD-1 receptor and induces the proliferation of regulatory T cells proliferation. TGF-β is another component of MSC-EVs, which also activates the formation of regulatory T cells (Chen et al., 2003).

The MSC-EVs have been shown to exhibit immunomodulatory properties *in vivo*. For example, it was shown that EV-treated mice demonstrated lower white blood cells (WBCs) counts and decreased neutrophil and monocyte influx into the hearts after myocardial ischemia reperfusion (MI/R) injury as compared to controls (Arslan et al., 2013). Also, MSC-EVs were able to switch the macrophages from a pro-inflammatory (M1 macrophages) to an anti-inflammatory (M2 macrophages) in the cardiomyopathy mice model and reduced the secretion of pro-inflammatory cytokines such as IL-1, IL-6, and TNF-α (Sun et al., 2018). The immunosuppressive effects of MSC-EVs on the liver injury animal models were also reported. It has been shown that the expression of pro-inflammatory cytokines such as IL-1, IL-2, TNF-α, IFN-γ was reduced while anti-inflammatory cytokines including TGF-β and HGF, and the number of T regulatory cells increased in the liver tissue following treatment with MSC-EVs (Tamura et al., 2016).

#### Mesenchymal Stem Cell Therapy for COVID-19 and the Other Viral Infections

Coronavirus disease 2019 (COVID-19) is a life-threatening infectious disease caused by a newly emerged coronavirus named severe acute respiratory syndrome coronavirus 2 (SARS-CoV-2). Patients infected with this virus will experience mild to severe respiratory disease (Rezasoltani et al., 2020a). There are currently no specific antiviral treatments licensed for COVID-19, however many treatments are under investigation (Rezasoltani et al., 2020b; Shpichka et al., 2021). Hopefully, the management of severe acute respiratory infection form of COVID-19 significantly can decline the death rate, particularly within the high-risk people. Several preclinical and clinical studies have exhibited the effects of exosomes and MSC-EVs in decreasing cytokine storm-associated complications, such as alveolar inflammation, edema, and epithelial tissue regeneration in inflammatory diseases, such as chronic obstructive pulmonary disease (COPD), asthma, acute respiratory distress syndrome (ARDS), and acute lung injury (ALI) (Akbari and Rezaie, 2020; Keller et al., 2020). Recently, MSCs-based immunomodulation therapy has been suggested as an effective treatment option for COVID-19 and multiple clinical trials have been launched so far (Akbari and Rezaie, 2020; Golchin et al., 2020). Since it has been suggested that the therapeutic effects of MSCs are essentially due to their secreted EVs, clinical trials may begin to apply MSC-derived exosomes and their EVs to alleviate the cytokine storm in severe COVID-19 patients (Akbari and Rezaie, 2020). However, there are some concerns over the safety, efficacy, and scalability of clinical-grade MSC-EVs (Golchin et al., 2020).

#### Secretion of Antimicrobial Peptides and Proteins

Associated molecular patterns are a miscellaneous class of naturally occurring small effector molecules that play a key role as the first line of defense by all multicellular organisms. AMPs can have wide killing activity against different types of microorganisms and even cancer cells. These biomolecules can also be referred to as ‘host defense peptides’, highlighting their additional immunomodulatory functions. Such functions are diverse, unique to AMP type, and involve a number of growth factor-like and cytokine effects that are contributed to normal immune homeostasis status (Seyfi et al., 2020). Some studies showed that MSCs elicit potent antimicrobial activities via indirect and direct mechanisms, partially mediated by the production of antimicrobial peptides and proteins (AMPs) of members of the cathelicidins, defensins, hepcidin, or lipocalin families as discussed below (Krasnodembskaya et al., 2010; Sung et al., 2016; Alcayaga-Miranda et al., 2017).

##### Cathelicidin LL-37

Cathelicidin LL-37 is the C-terminal part of the host cathelicidin, called human cationic antimicrobial protein (hCAP18), which is mostly produced by epithelial cells and neutrophils. The cathelicidin hCAP18/LL-37 is a multifunctional molecule that may regulate different human cellular and molecular processes such as epithelial cell activation, chemotaxis, bactericidal function, angiogenesis, and activation of cytokine and chemokine production. This antimicrobial peptide is produced from host cells upon infection of mycobacteria and exerts a bactericidal activity (Sandra Tjabringa et al., 2005). Besides a broad range of antimicrobial activity, LL-37 shows multiple immunomodulatory effects, anticancer functions, and also pro-angiogenic and chemotactic features. LL-37 has been found in many types of body fluids, tissues, and cells, and along with AMPs plays a critical role in host mucosal defense against microbial infections (Alcayaga-Miranda et al., 2017).

##### Human β-defensin-2

The hBD-2 is a cysteine-rich, cationic, low molecular weight antimicrobial peptide that is predominantly microbicidal against Gram-negative bacteria. It is expressed by many epithelial cells, granulocytes, and MSCs (Alcayaga-Miranda et al., 2017). The hBD-2 is a remarkable, inducible, antimicrobial peptide in a variety of epithelial cell types including skin cells, airways, kidney, oral mucosa, and gastrointestinal tract (Harder et al., 2000; Kumar et al., 2006). Its production is also induced by pro-inflammatory stimuli such as TNFα or microorganisms. The hBD-2 serves as a dynamic part of the local epithelial defense system of respiratory tract and skin which protect surfaces from infection. This is the reason why lung and skin infections caused by Gram-negative pathogens are rather rare (Li et al., 2016). Thus, based on significant antimicrobial and antiviral functions, modulating endogenous production of defensin by certain regulatory factors makes them promising therapeutic options against microbial infections.

##### Hepcidin

Hepcidin is a peptide encoded by the *HAMP* gene in human and is a natural host defense peptide found in urine (Nikfarjam et al., 2020) and plasma (Babaei and Rezaie, 2021). This peptide produces mainly by hepatocytes but other cells such as MSCs and myeloid leukocytes are also produce and release these peptides (Balhuizen et al., 2021). Two forms of hepcidin peptide, hep-20 and hep-25, exhibit antimicrobial properties (Nikfarjam et al., 2020) but hep-25 (LEAP-1) is also involved in the iron regulation (Chronopoulos and Kalluri, 2020). Beyond the iron regulatory effects, hepcidin has a broad spectrum of antibacterial and antifungal activity. For example, the antibacterial activity of this peptide against *Escherichia coli*, *S. epidermidis*, *S. aureus*, and group *B streptococci* has been shown previously which demonstrates its role as an antimicrobial peptide (Bitto et al., 2017). Incorporation of hepcidin into the EVs derived from the hepatocytes, MSCs, and myeloid leukocytes can be a mechanism for the prevention of microbial diseases.

##### Lipocalin-2

Lipocalin-2, also known as neutrophil gelatinase-associated lipocalin (NGAL), siderocalin, or 24p3, is a protein mainly secreted by neutrophils in response to infection and inflammation (Deng et al., 2020). Lnc2 blocks the siderophore iron-acquiring strategy of bacteria which leads to bacterial growth inhibition. It was shown that Lcn2-deficient (Lcn2^–/–^) mice were more sensitive than wild-type mice to bacterial infection (Nolte-‘t Hoen et al., 2016; Urbanelli et al., 2019). Moreover, Lcn2 is one of the components of the innate immune response against bacterial infection (Deng et al., 2020). MSCs are able to produce the Lnc2 protein and upregulation of this protein is directly corrected with bacterial clearance. Administration of antibodies against the Lnc2 protein have been found to block antimicrobial effects of MSCs (Riley et al., 2013).

##### Indoleamine 2, 3-dioxygenase

Mesenchymal stem cells secrete several soluble factors, including indoleamine 2, 3-dioxygenase (IDO). IDO is a tryptophan-degrading enzyme with antibacterial properties. IDO is involved in the antibacterial defense of some human cells and this was shown by using IDO specific inhibitors or by antagonizing the antibacterial effect with supplemental tryptophan. This enzyme acts against both intracellular (especially Chlamydia species) (Golchin et al., 2020; Dabrowska et al., 2021) and extracellular bacteria such as *Staphylococcus aureus*, *Streptococcus suis*, *enterococci*, and group *B streptococci* (Sandra Tjabringa et al., 2005; Alcayaga-Miranda et al., 2017). EVs derived from MSCs and containing IDO might be able to fight microbial diseases and reduce their growth rate *in vitro*.

##### Interleukin-17

Interleukin-17 (IL-17), a pro-inflammatory cytokine, contributes to host defense against both extracellular and intracellular pathogens. The antibacterial properties of this cytokine against *Aspergillus fumigatus*, *Cryptococcus neoformans*, and *Candida albicans* were demonstrated (Lombardi et al., 2015; Wang Q. et al., 2019). A variety of cells including CD4^+^ Th17 cells, CD8^+^ T cells (Tc17), natural killer T (NKT) cells, macrophages, and IL-17^+^ MSCs have the capacity to produce IL-7 (Yang et al., 2013; Mardpour et al., 2018; Duong et al., 2019; Shpichka et al., 2021). The IL-17^+^ MSCs are able to inhibit the growth of *C. albicans in vitro* and have a therapeutic effects on *C. albicans*-infected mice (Schroten et al., 2001). EVs derived from these MSCs, can inhibit bacterial growth.

### Potential Application of Stem Cell-Derived Extracellular Vesicles on Pathogenic Microbes

#### Extracellular Vesicles as a Unique Drug Delivery System

**FIGURE 2:**
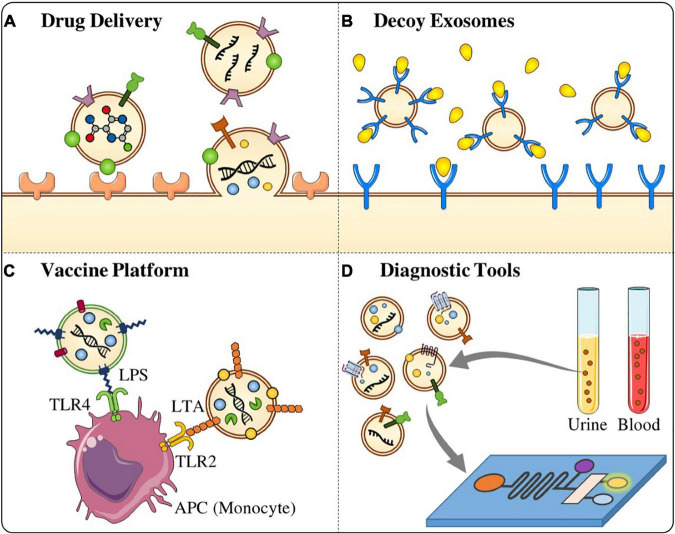
Potential application of stem cell-derived EVs on pathogenic microbes. **(A)** Stem cell-derived EVs can be used as a targeted drug delivery tool against infectious microbes. **(B)** Decoy exosomes are a biological trap which can absorb and antagonize detrimental factors such as bacterial toxins and inflammatory mediators. **(C)** EVs derived from MSCs are also can be used as vaccine platform to activate the immune response and react against the infectious diseases. **(D)** MSCs-derived EVs are good candidate for the development of diagnostic tools as they are involved in several biological processes and isolated from different biofluids.

Targeted drug delivery is among the most significant challenge in pharmacology and pharmaceutical sciences (Nolte-‘t Hoen et al., 2016). Distinctive properties of EVs favor their utilization as novel DDSs over synthetic ones (Figure 2). These characteristics include their capability to cross physical barriers, their biocompatibility, their inherent targeting features, and also their ability to exploit natural intracellular trafficking pathways (Elsharkasy et al., 2020). Interestingly, viruses incorporate specific binding proteins, and thus are highly targeted mostly due to their evolved and acquired high specificity toward their cellular targets. The EVs membrane can be engineered to incorporate with such specific viral proteins to facilitate EV-mediated transfer of drugs (György et al., 2015). Also, genetically manipulated cells from which EVs are originated, have been developed to provide distinct platforms for loading cargo and conjugation of targeting moieties to their EVs. However, further in-depth investigations into EV biogenesis, EV subpopulations, cargo sorting, internalization and trafficking routes in recipient cells are required to achieve translational applications of such engineered EVs. Furthermore, there are a number of obstacles that should be addressed toward clinical use and include scale-up of the EV production and isolation process, as well as standard protocols for proper banking (Elsharkasy et al., 2020).

#### Decoy Exosomes

Decoy exosomes represent a new class of therapeutic biologics that are generated by molecular engineering approaches to treat human diseases including inflammation, cancer, and cardiovascular disorders (Zhang et al., 2021). This type of exosomes functions as a biological sponge to absorb and antagonize detrimental factors such as bacterial toxins and inflammatory mediators particularly, TNFα, in host blood or tissues (Figure 2; Duong et al., 2019). Nonetheless, the scale-up production of decoy exosomes from more suitable producing cells is essential to obtain high-quality exosomes for therapeutic utilization against infections and inflammatory diseases.

#### Extracellular Vesicles as Vaccine Platform

Extracellular vesicles from pathogenic bacteria usually carry PAMPs and MAMPs which authorize them to activate the immune response, macking them the ability to be applied as vaccine candidates (Figure 2; Sharpe Samantha et al., 2011; Gorringe and Pajón, 2012; Bartolini et al., 2013; Mehanny et al., 2020). For instance, the BEVs from *Neisseria meningitides*, have been applied as the basis for a vaccine against meningococcal disease, as they induce antibacterial immune responses (Gorringe and Pajón, 2012). The outstanding outcomes of BEVs-based vaccines demonstrated a new avenue and proposed novel strategies to immunize individuals against pathogenic bacteria (Gorringe and Pajón, 2012; Fantappiè et al., 2014; Shkair et al., 2021). Moreover, EVs derived after viral inoculation may further be applied to develop more effective vaccines against viral infections by adding or expelling certain subpopulations of them. In contrast to utilizing pathogenic BEVs for vaccine targets, it has been proposed that BEVs originated from symbiotic bacteria may exhibit modulatory effects on host immune system. For instance, *Bacteroides fragilis* can selectively deliver capsular polysaccharide A (PSA) cargo in its BEVs that have been shown to induce immunomodulatory responses and prevent colitis in mice. These data support the rationale for designation of novel probiotic formulations based on specific beneficial BEVs which can be used for therapeutic purposes (Muraca et al., 2015; Choi et al., 2017).

#### Cell-Derived Extracellular Vesicles as Diagnostic Tools

Extracellular vesicles are involved in several biological processes and isolated from different biofluids which make them valuable biomarkers for the early diagnosis or prognosis of various diseases such as cancer, inflammatory diseases, and infections (Figure 2; Wei et al., 2021). Thus, these vesicles can be regarded as interesting and non-invasive biomarkers for the diagnosis of different diseases (Wei et al., 2021). EVs isolated from the blood have gained significant interest mainly in the context of tumor diagnosis, and their fluctuations are associated with tumor progression, metastasis, and immune evasion (Palmirotta et al., 2018; Nikfarjam et al., 2020; Salimi et al., 2020). Glypican-1 (GPC1), for instance, as a cell surface proteoglycan, is specifically expressed by exosomes isolated from the serum of pancreatic cancer patients, and it is used as an early biomarker. Further, it has been reported that levels of GPC1^+^ exosomes are correlated with pancreas tumor burden and survival rate (Melo et al., 2015). EVs also can be used for the detection of early stages of metastasis. For example, in the exosomes of patients with metastatic melanoma, MDA-9 and GRP78 proteins have higher expression than those of patients without metastases (Guan et al., 2015). Moreover, EVs as urinary biomarkers have been introduced for the early diagnosis of a variety of kidney and genitourinary tract disorders (Street et al., 2017). Neutrophil gelatinase-associated lipocalin (NGAL) (Alvarez et al., 2013), polycystin-1 (PC1) (Hogan et al., 2015), transmembrane protein 2 (TMEM2) (Hogan et al., 2015), and WT-1 (Wilms’ tumor-1) (Zhou H. et al., 2013) are some exosomal biomarkers that can be applied for the diagnosis of renal diseases. Some exosome/EV products are commercially available for the diagnostic purposes. For example, ExoDX Lung (ALK), the world’s first exosome-based diagnostic kit, was developed and passed FDA certification in Wei et al. (2021).

As aforementioned, EVs have been considered as reliable biomarkers in the context of infectious diseases. From a diagnostic point of view, EVs carry antigens from parental cells and act as reporters of foreign agents (Yáñez-Mó et al., 2015). In the *M. tuberculosis*-infected patients, mycobacterial proteins responsible for *M. tuberculosis* intracellular survival were identified from their secreted exosomes (Kruh-Garcia et al., 2014). It was shown that the mRNA (Lv et al., 2017) and miRNA (Lyu et al., 2019) profiles of exosomes derived from the sera of healthy cases and patients with active and latent tuberculosis were different, which could be used as a diagnostic biomarker. Moreover, human macrophages infected with *Yersinia pestis* and *Bacillus anthracis* secrete particular miRNA-containing exosomes (Fleming et al., 2014). LPS induces the murine bone marrow-derived dendritic cells (BMDC) to secrete exosomes containing miR-146a and miR-155 (Jiang et al., 2019). *Helicobacter pylori* infection also exhibits an increase in miR-155 level in the exosomes derived from macrophages (Wang et al., 2016). These miRNAs are proper biomarkers for the rapid detection of such infectious diseases.

## The Driver Challenges for the Application of Stem Cell-Derived Extracellular Vesicles in Clinic

**FIGURE 3:**
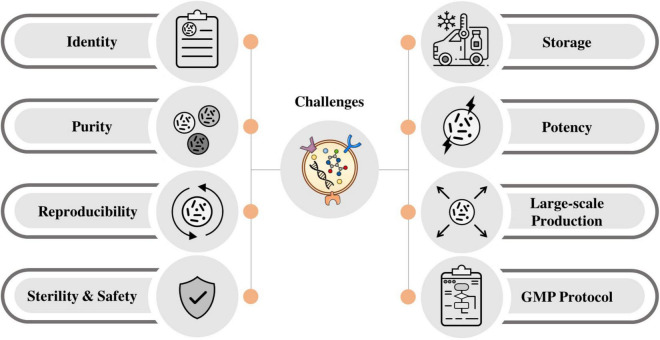
Main challenges for the application of stem cell-derived EVs in clinic.

The process of manufacturing MSC-EVs in the clinic needs donor identification and screening. Donor identification and screening need a complete review of risk factors, relevant communicable disease agents, and diseases as outlined in FDA 21 CFR Donor Screening 1271.75. Common sources of MSCs and then EVs for the clinical application are those derived from bone marrow and adipose tissue. The EVs harvested from these tissues are then further characterized for their identity, purity, potency, and sterility. Currently, several challenges for manufacturing the clinical-grade EVs are ongoing, and many modifications and optimizations are needed to ensure the safety and reproducibility of the EVs as therapeutic agents (Wiest and Zubair, 2020). Some of the challenges are briefly described below (Figure 3).

### Identity

Due to their nanometer to micrometer scale, detection of EVs by means of currently available lab equipment is challenging. Also, the tissue sources of the MSCs have an impact on the EVs characteristics. It was shown that the origin of the MSCs has an impact on the amounts and sizes of EVs. Exosome samples isolated by ultracentrifugation (UC) and tangential flow filtration (TFF) showed abundant expression of CD81 and CD9 markers and depletion of calnexin as compared to parental cells, but CD63 marker was only expressed on UC-isolated EVs (Haraszti et al., 2018). Therefore, different isolation methods produce different populations of EVs, and thus emphasize the need for standardized good manufacturing practices (GMPs). Because of different components of EVs, some databases such as ExoCarta, Vesiclepedia, and EVpedia have been established in recent years (Keerthikumar et al., 2016; Pathan et al., 2019). These databases have valuable resources for the identification of different EVs, but some specific and universal markers for each EV have not yet been provided. Therefore, further studies are required for the precise characterization of EVs and determining their identity.

### Purity

Since EVs are derived from different cells, including MSCs, they may contain some impurities. For example, fetal bovine serum (FBS) is often added to the MSCs culture media. It was shown that FBS fractions contain RNA molecules and deep sequencing of these fractions showed that 13.6% and 21.7% of the RNA in cell pellet and supernatant mapped to the human genome, respectively (Wei et al., 2016). Some laboratories employed the UC for the removal of the majority of EVs and miRNAs found in the FBS (Shelke et al., 2014; Wei et al., 2016). Because of xenobiotic contents of FBS, some manufacturers used human platelet lysate instead of FBS. Human platelet lysate is xenobiotic-free and fibrinogen-depleted and can be used for MSCs culture in GMP studies. It was shown that UC could be able to eliminate the serum-derived RNAs in the lysates (Pachler et al., 2017b). It should be noted that EVs isolated by UC and anion exchange chromatography have similar markers and size distribution, but EVs enriched by TFF are not similar (Heath et al., 2018). Other methods were developed to improve the EVs’ purity include quantification of protein to particle ratio (Webber and Clayton, 2013) and protein to lipid ratio (Osteikoetxea et al., 2015); however, these methods still have some limitations.

### Reproducibility

Many factors influence the content and amount of EVs released by cells and there are currently no standard protocols for the isolation and storage of EVs (Lener et al., 2015). Also, there is MSC donor-to-donor and batch-to-batch variation (Russell et al., 2018). It was shown that EVs are highly sensitive to cell stress, and their content may be changed in response to stress. For example, treatment of human placental cells with tunicamycin induces ER stress in these cells and leads to the release of EVs containing HSP70 and HMGB1 (Collett et al., 2018). The content of the cell culture media, the composition of the serum added to the media, and the drug interactions affect the EVs’ integrity (Wiest and Zubair, 2020). Isolation methods also change the composition of EVs. Haraszti et al. showed that when MSCs were grown in 3D culture conditions and isolated by TFF, displayed a different protein content compared to other methods of culturing and isolation (Haraszti et al., 2018). Most studies focus on UC as a gold standard for the isolation of EVs. Busatto et al. (2018) reported that when they used the US for isolation, the size distribution and albumin purity of samples changed significantly from batch-to-batch, but using TFF, in contrast, showed less batch-to-batch differences. These studies highlighted the factors affecting the reproducibility of EVs and emphasized the need for the development and validation of isolation methods.

### Sterility and Safety

Sterility tests are assays performed by manufacturers to determine the microbial contaminations. Strict donor eligibility criteria and screening methods for diseases are the first steps for the determination of sterility and safety of the products. The companies generating EVs must comply with FDA Title 21, Part 610 (Wiest and Zubair, 2020). Microbial contamination is important not only for the safety of the recipients, but also some microbes produce and secret EVs which might interfere with the EVs in the therapeutic product (Quah and O’neill, 2007; Giri et al., 2010). The size of some viral particles is similar to EVs which raises some challenges for EV isolation by size-dependent techniques such as TFF and other chromatography-based methods (Matthews, 1975). TFF is better than UC for reducing the risk of contamination because TFF method can be conducted in a closed system, but UC technique requires multiple steps and requires multiple transfer of the fractions to the new containers (Wiest and Zubair, 2020).

Due to cell-free nature, EVs are hypothesized to be safer than other products, but there are limited data about the safety profile of EV-containing compounds (Wiest and Zubair, 2020). A short-term safety study was conducted by Montaner-Tarbes et al. (2018), where they treated healthy pigs with EVs derived from pigs with the porcine syndrome. They found that healthy pigs treated with EVs have no signs of the disease (Montaner-Tarbes et al., 2018). Until recently, adverse events (AEs) or toxicity related to EVs-based treatments are rarely reported (Nassar et al., 2016; Saleh et al., 2019).

### Storage

Extracellular vesicles are very sensitive to temperature and pH of the storage buffer (Wiest and Zubair, 2020). The ideal temperature for the long-time storage of EVs is –80°C (Otrokocsi et al., 2014). It has been shown that the quantity of EVs decreases in a time-dependent manner when they are stored at room temperature or 4°C following isolation. Also, the results of the light-scattering analysis demonstrated a notable time-dependent increase in the structural changes of EVs when they stored for a long time at –20°C (Otrokocsi et al., 2014). Changes in the pH of the storage buffer can induce EVs’ aggregation and loss of their functionality (Lener et al., 2015). The storage buffer is also an important factor for maintaining the EVs’ functionality. The phosphate-buffered saline was exploited in the majority of published studies as the EVs storage buffer (Li et al., 2019; Marcoux et al., 2019), but others also have used sucrose buffer (Busatto et al., 2018), lactated Ringer’s solution (Pachler et al., 2017b), and PBS supplemented with trehalose (Bosch et al., 2016) for the storage of EVs. The type of EVs and their application may require different storage buffers and maintaining conditions.

### Potency

Despite the difficulties in identifying active components in the EVs, potency determination becomes more popular in the last few years in preclinical studies (Wiest and Zubair, 2020). Due to the pleiotropic effects of proteins and RNAs contained within EVs, identifying the active ingredients in exosome therapy is challenging. EVs’ potency assays are promising methods to overcome the challenges of identifying an active ingredient. The basics of many potency assays is the release of pro-inflammatory cytokines by M1-phenotype macrophages. To do this, EVs must be added to the culture of M1-phenotype macrophages and the desired inflammatory marker is measured based on the dose of EVs (Pachler et al., 2017a; Willis et al., 2018). These methods are used frequently for the potency evaluation but further research is needed to validate their applications.

### Large-Scale Production

Large-scale production of EVs for clinical applications needs scale-up culture of MSCs, but long-term passaging may result in losing clonal and differentiation capacity of cells (Raghav et al., 2021). Therefore, it is necessary to develop new methods for reliable expansion of MSCs to mass-produce EVs for clinical use. Also, large-scale culture of MSCs in bioreactor requires the addition of some ingredients such as fibronectin for cell adherence purposes. Fibronectin as an ingredient has some complications because this protein makes clogging in filter pores and interferes with the size-based selection of EVs using the TFF method. Therefore the conditioned media need an extra centrifugation step, which make the risk of contamination more (Wiest and Zubair, 2020).

### Developing a Good Manufacturing Practice Protocol

For the production of EVs in large quantities, the companies demand a standardized manufacturing process which must comply with GMP regulations (Mobarak et al., 2021). The GMP-grade production of EVs is a process that depends on the cell type, culture media, cultivation, and purification methods. In the case of cell type, five cell types including bone marrow and adipose tissue-derived MSCs, monocyte-derived dendritic cells (DCs), human cardiac progenitor cells, and HEK293 cells have been used in GMP-grade for production of EVs. Cultivation methods employ both static and dynamic systems. Flask based systems are static but bioreactor systems are dynamic (Rezaie et al., 2021). In the GMP-grade production of EVs, bioreactor systems are preferred due to the dynamic monitoring system (Akbari and Rezaie, 2020). In the case of purification of GMP-grade products, a number of steps including filtration for the removal of cell debris, centrifugation for enrichment of the conditioned media, and isolation of EVs from the media should be performed. Differential centrifugation, despite its complications, is the preferred method for concentrating the conditioned media (Salimi et al., 2020).

## Conclusion

In recent years we have witnessed remarkable progress in the biology of EVs and their impact on microbial diseases. Now, a clear picture has emerged and showed that MSCs-derived EVs may play a crucial role in infectious diseases. MSCs-derived EVs retain the biological activity of parental MSCs and have a similar therapeutic potential. Evs derived from MSCs have potent antimicrobial activity by production of antimicrobial peptides and proteins (AMPs) such as hepcidin, lipocalin, defencins, etc. Also, MSCs-derived EVs are applicable in drug delivery systems, vaccine platform, and diagnostic tools to fight infectious diseases. In clinic, several challenges exist for the manufacturing clinical- and GMP-grade EVs which needs to be addressed. Further understanding of the manufacturing of EVs for clinical application, their biogenesis method as well as their optimization method can reduce many of the challenges in using MSCs-derived EVs in the clinic. Taken together, these data suggest that MSC-derived EVs could be promising therapeutic tool for the treatment of infectious diseases.

## Author Contributions

HK involved in drafting and editing. BS involved in drafting and figures. SR and NH-K involved in drafting. AY and MH involved in conceptualization, editing and proofreading. MV involved in conceptualization, reviewing and editing the draft, and final approval. All authors contributed to the article and approved the submitted version.

## Conflict of Interest

The authors declare that the research was conducted in the absence of any commercial or financial relationships that could be construed as a potential conflict of interest.

## Publisher’s Note

All claims expressed in this article are solely those of the authors and do not necessarily represent those of their affiliated organizations, or those of the publisher, the editors and the reviewers. Any product that may be evaluated in this article, or claim that may be made by its manufacturer, is not guaranteed or endorsed by the publisher.
